# Medical News and Miscellany

**Published:** 1892-05

**Authors:** 


### Medical News and Miscellany.

Married.— Dr. Byron F. Kingsley to Miss Nellie A. Glennon, both of San Antonio, Tuesday, April 26th ult.

Dallas, Texas.—At an election held on the 9th inst., Dr. C. M. Rosser, City Physician, was defeated by Dr. V. P. Armstrong.

Wanted to buy.—A location and established practice in some good section in Texas. Address: "Magnum," care DANIEL'S TEXAS MED. JOURNAL, Austin, Texas.

Texas Students.—Texas furnished 493 medical students last year. Read Dr. Paine's speech,—the statement is made on authority of Illinois State Board of Health.

Dr. B. A. Harris, formerly of Oklahoma, has just returned from St. Louis with a diploma from the Marion Sims, and has located at Harold, Wilbarger county, for practice.

Prof. Wm. Keiller, the anatomist of the Texas Medical College, sailed for Europe on the 5th inst. His home is in Edinburgh. All the professors, except those whose home is there, have left Galveston for the summer.

A Paris Correspondent of the *Lancet* (says the New York *Medical Record*), tells of a surgeon who performed laparotomy, and left a gauze compress in the cavity, which compress enveloped in a mass of hard feces was passed per rectum.

**Dr. G. W. Christian**, of Burnet, Texas, has been called to the City of Mexico to perform a gynecological operation. This is, indeed, a feather in the doctor's cap. He passed through Austin on the 10th inst., and will return in about two weeks.

---

**Cerebral Localization.**—The Dios Chemical Company, St. Louis, have published a colored chart, showing the location of all the motor centres in the human brain; a copy of which will be mailed to any physician free on application mentioning this journal.

---

**Prof. J. E. Thompson, M. D.**, of the Texas Medical College, of whose illness the JOURNAL made mention last month, we are glad to say, is now convalescent, and still in Austin. He will shortly sail for his old home in Manchester. He will return in September.

---

**Dr. J. Monroe Richmond**, of Manor, Texas, has returned from St. Louis with his diploma from the Marion Sims Medical College, after a three years course. The JOURNAL acknowledges the courtesy of an invitation at his hands to attend the commencement exercises.

---

**Dr. Grandstaff**, of Mason, Texas, is at the Philadelphia Polyclinic. This school is popular with Texans, because the classes are never allowed to get so large as to be unhandy; every matriculant receives personal attention and instruction. Write to Dr. Baldy, the Dean, about it.

---

**Dr. E. M. Rabb**, of Hallettsville, the JOURNAL regrets to learn, from overwork and study, was recently the victim of cerebral apoplexy and formation of a blood-clot in the brain. As soon as he was able to travel he went to Hot Springs, Ark., where, we learn, he has about recovered. The Doctor has gone to New York, and is taking a special course at one of the Post-graduate Colleges.

---

**Substitution Squelched.**—The United States circuit court of Louisiana, in a case entitled Battle & Co. Chemist Corporation (the well-known manufacturers of Bromidia), vs. Finley & Brunswig, decided that the latter had infringed upon the rights of Battle & Co., in the use of the word "Bromidia," and issued an injunction restraining them from the further use of the word, as applied to their own preparations. Served them right.

---

Dr. M. L. Perry, of Lancaster, Texas, who graduated at the University of Nashville Medical College last year, won the highest hospital honor in the city. This popular college had last session the largest class in its history, and of the number Texas furnished seventy. The Texas students are most always prize winners, or press very closely those who are. Send to Dr. Eve for a catalogue and read announcement in this issue.

---

The Nedofik.—The Nedofik sofa, or chair, is advertised herein. It is a masterpiece of ingenious mechanism, and the embodiment of comfort and convenience; can be put into any position in which a surgeon, gynecologist, dentist or lazy lounger may want to use it. Doctors should investigate it, and appreciate its merits. There is Nodefik-ulty in its manipulation; can be worked by any one, and is ornamental as well as useful.

---

There will be a meeting of the Association of American Medical Colleges in Detroit, June 8, during the session of the A. M. A. Dr. Davis will have a paper on the extent of Chemical Instruction that should be afforded a student of medicine in his regular course, and Dr. Vaughn one asking the same conundrum as to laboratory work. We anticipate by answering—all that time and opportunity allow; didactic instruction is a badly motheaten back number; musty.

---

Our own Dr. W. P. Willis, of Montgomery, Texas, exhibited at the Tyler meeting of the Texas State Medical Association the cranial and other bones of a fetus, which had been expelled per rectum from one of his patients, in whom he had diagnosed extra-uterine pregnancy thirteen months previously. He was not permitted to operate at the time, nor subsequently; meantime, he says, patient fell into the hands of another doctor, who treated her for cancer.

---

Prof. Seth M. Morris, M. D., the chemist of the Medical Department of the University of Texas, left Austin for New York via Galveston on the 9th inst., thence he will sail on the 20th for Bremen. He goes to Berlin to spend the college vacation in attendance upon the great schools there, taking a special course in physiological chemistry. While in Europe Prof. Morris, as the accredited agent of the Regents, will purchase and ship in time for the next session a complete laboratory outfit.

---

**Removals.**—Dr. L. F. Layton, late of Lampasas, who removed to Beeville, has changed his address again and is at San Diego, Texas. Dr. Wm. Osborn has removed from New Birmingham to Waco.

Mr. Robt. P. Daniel, son of Dr. F. E. Daniel, has been transferred from Chihuahua, Mexico, to Tampico, Mexico. He is in the service of the Mexican Central R. R. Hospital Department, and has been stationed at Chihuahua with Chief Surgeon Paschal some three years.

---

"**American Men of Eminence**" is the title of a neat little "brochure" recently sent us by the ARLINGTON CHEMICAL CO., of Yonkers, N. Y., the well-known manufacturers of peptonoids. Amongst the portraits of eminent men that of the late Dr. Fordyce Barker is conspicuous. It is simply *grand*. It is the finest piece of engraver's art we ever saw, and is well worth framing and preserving in any physician's office. The Arlington Chemical Company will send this book free of charge to any physician who asks for it, naming this Journal.

---

THE AUSTIN DISTRICT MEDICAL SOCIETY.—The next quarterly meeting of this Society will be held on the 23d of June, in Medical Hall, Austin. The usual number of good papers will be read and discussed, and other business of importance transacted. The Central Texas Medical Association has invited the Austin District Medical Society to attend a joint meeting, in Waco, on the 14th of October. The full programme of the joint meeting will be published in the TEXAS SANITARIAN in due time. Let us have a full attendance from the district on June 23d. The Austin District boasts of being the banner Society of the State.

---

**Witty (?).**—For new (?) and original wit, several centuries old, commend us to the St. Louis *Weakly*. The May 14th issue had that rich joke about Mrs. Partington having two buckles in her lungs, and the college graduate with two diplomas likened to the calf which sucked two cows, and was nothing but a calf yet. We nearly laughed our poor self to death. Where on earth has that editor been all his life that he never heard those stale old jokes? Well, he has to fill up on something, and we having objected to his stealing any more of this journal's editorials, we suppose he is in straights. Brother *Weakly*, try to *think* of something; you can do it with a little practice, maybe.

---

The Texas Medical College had but twenty-two matriculants, and graduated three the first session (closed April 22, ult.) This may seem a small beginning, but when it is remembered that an examination for matriculation is required, a three year's graded course, a high curriculum, and fees as high as the highest, it is indeed a flattering beginning; especially as these gentlemen could easily have matriculated at some quite reputable college without examination, and, for a much smaller fee, have completed their course in two years, and come away with a *diploma* which, practically, will answer every purpose, and admit to any Medical Society.

---

A Memorial Edition.—Look out for our July number—our next issue, June, '92, will complete Vol. 7, and the JOURNAL'S seventh year of success. This important event will be commemorated by beginning Vol. 8 with a double edition—2000 copies, with the size of the page and number of reading pages increased. The July number (No. 1, Vol. 8) will be illuminated by half tone engravings of the Medical Faculty of our own Texas Medical College, the Medical Department of the University of Texas, and also handsome cuts of the College building and the Sealy Hospital. It will contain some interesting reading in connection with the College, and biographies of the several professors. It will be a memorial volume, and every physician who feels a pride in the successful inauguration of a high grade Medical College in Texas should secure a copy for preservation.

---

"Crocodile Tears." —The St. Louis *Weekly Medical Review*, noticing the *Texas Sanitarian's* accusations of piracy against the *Northwestern Medical Journal*, says *hinc illae lachrymœ.* While we do not see the appropriateness of the quotation, the tears may be there; and if so, are evidently crocodile tears, for in the same issue we find an editorial paragraph stolen from DANIEL'S TEXAS MEDICAL JOURNAL, appropriated without credit, and passed off as *original.* (See page 377, "Exit Prof. Amick.") In another place we have observed a similar piracy; one for which the *Weekly Review* has received a good deal of notice. "The Ladies' Friend" article first appeared in this journal, and being appropriated by the *Weekly Review* without credit, is being copied all around and credited to *that journal;* the funniest part being, it is credited to "H. M. Lewis, in the *Weekly Review,*" whereas "H. M. Lewis" was the she-fiend who sold the so-called "recipe" which we published. Come now, St. Louis *Weekly Review*, if you are too lazy to get up original editorial matter, and find the Red-Back a good labor-saver, give us credit for our squibs, at least, won't you?

---

**A Wooing to the Woods.**— We are something of a disciple of Isaak Walton, and love nothing so much as to lure the festive trout from his haunts with the deceptive fly; and when the spring time comes,—when "the young man's fancy lightly turns to thoughts of love,"—*our's* turn to the pleasures of the rod and reel. The temptation is strong at this season; but when, from a brother M. D., who is blessed with the world's goods, a delightful home, an interesting family, a big practice, and a heart as open as a Southern sky, and as generous as its breezes; himself a skilled angler, whose home is in the most picturesque portion of picturesque Southern Texas,—on the banks of the classic Comal,—couched in the language of the poet, the temptation is irresistible, and we yield. The Doctor writes:

> "Its just the time for dreamin' of the cool and shady nooks;
> For rolling up your breeches for a splash into the brooks.
> Its wishin' time, its fishin' time, its time to take your ease
> Where the locust sings soprano to the tenor of the bees.
> Oh Editor! leave your ink-stand, an' your drowsy, frowsy desk,
> An' get out in the country where the world is picturesque!
> Oh! man, dead set for money; oh! toiler in the strife!
> Slip off and get some honey that will sweeten up your life."

"Come,—and bring Bennett and Rainey with you.

"Yours truly,

